# Lactic acidosis induces resistance to the pan-Akt inhibitor uprosertib in colon cancer cells

**DOI:** 10.1038/s41416-020-0777-y

**Published:** 2020-03-10

**Authors:** Emily M. E. Barnes, Yitao Xu, Adrian Benito, Lili Herendi, Alexandros P. Siskos, Eric O. Aboagye, Anke Nijhuis, Hector C. Keun

**Affiliations:** 1 Cancer Metabolism & Systems Toxicology Group, Division of Cancer, Department of Surgery & Cancer, Imperial College London, Hammersmith Hospital Campus, London, W12 0NN UK; 2 Cancer Imaging Centre, Imperial College London, Hammersmith Hospital Campus, London, W12 0NN UK; 3 Division of Systems Medicine, Department of Metabolism, Digestion & Reproduction, Imperial College London, Hammersmith Hospital Campus, London, W12 0NN UK

**Keywords:** Cancer metabolism, Cancer therapy

## Abstract

**Background:**

Akt signalling regulates glycolysis and drives the Warburg effect in cancer, thus decreased glucose utilisation is a pharmacodynamic marker of Akt inhibition. However, cancer cells can utilise alternative nutrients to glucose for energy such as lactate, which is often elevated in tumours together with increased acidity. We therefore hypothesised that lactic acidosis may confer resistance to Akt inhibition.

**Methods:**

The effect of the pan-Akt inhibitor uprosertib (GSK2141795), on HCT116 and LS174T colon cancer cells was evaluated in the presence and absence of lactic acid in vitro. Expression of downstream Akt signalling proteins was determined using a phosphokinase array and immunoblotting. Metabolism was assessed using ^1^H nuclear magnetic resonance spectroscopy, stable isotope labelling and gas chromatography-mass spectrometry.

**Results:**

Lactic acid-induced resistance to uprosertib was characterised by increased cell survival and reduced apoptosis. Uprosertib treatment reduced Akt signalling and glucose uptake irrespective of lactic acid supplementation. However, incorporation of lactate carbon and enhanced respiration was maintained in the presence of uprosertib and lactic acid. Inhibiting lactate transport or oxidative phosphorylation was sufficient to potentiate apoptosis in the presence of uprosertib.

**Conclusions:**

Lactic acidosis confers resistance to uprosertib, which can be reversed by inhibiting lactate transport or oxidative metabolism.

## Background

Historically, upregulated glycolysis (also known as the Warburg effect) was considered necessary to provide rapidly proliferating cancer cells with sufficient energy and biosynthetic intermediates.^1^ However, we now appreciate that cancer cell metabolism is far more flexible and heterogenous.^2^ For instance, tumours utilise mitochondrial metabolism and oxidative phosphorylation (OXPHOS)^3^ for energy production and anabolism. Furthermore, glucose concentrations within tumours are often low,^4^ therefore, cancer cells must adapt to survive under severe nutrient stress, often through utilisation of alternative nutrients, such as amino acids and lipids.^5–9^

Lactate is the end product of glycolysis and is often present at elevated concentrations within tumours (10–20 mM).^1,10,11^ Lactate transport via monocarboxylate transporters (MCTs) is bi-directional,^12,13^ thus cells can import lactate, to be used as a fuel for oxidative metabolism and gluconeogenesis.^3,14–16^ Moreover, preferential utilisation of lactate as a carbon source over glucose in the tricarboxylic acid (TCA) cycle has been suggested to occur in some human lung tumours.^15^ It is becoming increasingly apparent that the role of lactate in promoting cancer cell survival and progression has been underestimated.

Akt signalling is implicated in multiple mechanisms that upregulate glucose metabolism, thus, oncogenic Akt activity is considered a key driver of the Warburg effect in cancer.^17^ As a result, therapeutic targeting of Akt leads to reduced glucose uptake, which is considered a pharmacodynamic marker of Akt inhibition.^18^ Uprosertib (GSK2141795) is an ATP-competitive pan-Akt inhibitor in phase II clinical trials.^19–21^ Despite this, efforts to identify patients that are most likely to respond have been relatively unsuccessful, with most responders demonstrating tumour growth inhibition and stable disease, as opposed to disease regression.^22^ Since uprosertib treatment leads to reduced glucose uptake in vitro and in patient tumours,^19,23^ in the current study we investigated whether lactate oxidation could induce resistance to uprosertib. We report that lactic acidosis induces resistance to uprosertib in colon cancer cells, which is reversed upon abolition of MCT activity or therapeutic inhibition of OXPHOS.

## Methods

### Cell culture

The human colon cancer cell line, HCT116, was a generous gift from Dr Volker Arlt at King’s College London (London, UK). The human colon cancer LS174T cells, with either wild-type or MCT4 knockout^24^ were generously provided by Prof. Jacques Pouysségur at the University of Nice (Nice, France). Dulbecco’s Modified Eagle’s Medium (DMEM; Cat #A14430; Gibco, Grand Island, New York, US), which contains a sodium bicarbonate buffer, was used for routine culture and all experiments. For routine culture, DMEM was supplemented with penicillin/ streptomycin (100 μg/ mL), D-glucose (5.6 mM), L-glutamine (2 mM) and 10% foetal bovine serum purchased from Gibco (Grand Island, New York, US). Cells were maintained in a 37 °C, 5% CO_2_ humidified incubator. Cell lines were authenticated using short-tandem repeat DNA profiling by Public Health England and mycoplasma tested.

Prior to experiments, cells were gradually adapted to increasing lactate concentrations to reduce the potential for cellular stress and growth inhibition caused by exogenous lactic acid supplementation. To do this, 3 × 10^6^ cells were cultured in T150 flasks for 3 days in routine culture medium to produce conditioned medium containing lactate. Cells were subsequently plated in conditioned medium for 24 h before experimental conditions were applied.

### Sulforhodamine B assay

To investigate the cytotoxic effects of uprosertib (Selleckchem, Houston, Texas, US) Sulforhodamine B (SRB) assays were performed according to manufacturer’s instructions.^25^ Cells were plated into 96-well plates at a density of 4 × 10^4^ cells per well and incubated for 24 h. Media were subsequently changed to DMEM (0 h) supplemented with glucose (5.6 mM), glutamine (2 mM), 10% FBS, lactic acid (0–20 mM; Sigma-Aldrich, St. Louis, Missouri, US) and uprosertib (0–15 μM) or vehicle (0.1% DMSO (Sigma-Aldrich, St. Louis, Missouri, US). Test media were replenished every 24 h to control for changes in extracellular metabolite concentrations. The SRB assay was performed at 0 and at 72 h post initial treatment. Data were presented as the Log_2_ of the optical density (OD; 565 nm) at 72 h relative to the OD at 0 h.

### Cell counts

Cells were plated at a density of 1 × 10^5^ cells per well into 12-well plates and incubated for 24 h. DMEM supplemented with glucose (5.6 mM), glutamine (2 mM), 10% FBS, lactic acid (0 to 20 mM) and uprosertib (10 μM) or vehicle (0.1% DMSO) was subsequently added to wells. Media were replenished every 24 h. TrypLe Express (ThermoFisher Scientific, Massachusetts, US) was used to de-adhere cells from wells. Cells were counted using the Vi-Cell XR Cell Viability Analyzer (Beckman Coulter, Indianapolis, US) at 0 and 72 h after initial treatment.

### Caspase 3/7 and ATP measurements

Cells were plated at 4 × 10^4^ cells per well into 96-well plates and incubated for 24 h before media were replaced with test media (0 h) supplemented with glucose (5.6 mM), glutamine (2 mM), 10% FBS, lactic acid (0–20 mM) and uprosertib (5 or 10 μM) or vehicle (0.1% DMSO). Apoptosis was determined at 24 or 48 h post initial treatment using the Caspase-Glo 3/7 assay system according to the manufacturer’s instructions (Promega, Madison, Wisconsin, US). ATP was measured after 24 h of treatment using the CellTitre-Glo® 3-D luminescent assay (Promega, Madison, Wisconsin, US) according to the manufacturer’s instructions. Luminescence was measured using the CLARIOstar plate reader (BMG Labtech, Ortenberg, Germany) and readings were normalised first to cell density determined using an SRB assay performed in a parallel plate and second to the vehicle controls.

### Culture of 3-D spheroids

HCT116 cells were plated into 96-well Ultra-Low Attachment plates (Corning, New York, US) at a density of 1 × 10^3^ cells per well in 50 μL of media supplemented with glucose (5.6 mM), glutamine (2 mM), 10% FBS and lactic acid (0 or 10 mM) and incubated for 24 h to allow 3-D spheroids to form. Subsequently, 50 μL of test media containing uprosertib (0 to 15 μM) was added on top of the original 50 μL and spheroids were incubated for 72 h. Brightfield images of spheroids were obtained using the IN Cell Analyzer 2000 (GE Healthcare, Chicago, Illinois, US) using a 4 × 0.2 NA objective lens. Scale bars represent 100 μm and were added using Image-J software. To quantify spheroid viability after 72 h of uprosertib treatment, we used the CellTitre-Glo® 3-D luminescent assay (Promega, Madison, Wisconsin, US) according to the manufacturer’s instructions.

### Phospho-kinase array

LS174T cells were treated with uprosertib (10 μM) in the presence or absence of lactic acid (10 mM) for 1 h before cells were lysed and protein was examined using the Proteome Profiler Human Phospho-Kinase Array Kit (R&D Systems, Minneapolis, MN, US) according to the manufacturer’s protocol. Chemiluminescent signals were exposed using the Supersignal^TM^ West Pico plus chemiluminescent substrate (Thermo Scientific, Rockford, US) onto Amersham Hyperfilm^TM^ ECL films (GE Healthcare, Chicago, Illinois, US) using the Optimax X-ray film processor (Protec Healthcare, Oberstenfeld, Germany). Densitometry was performed using Image-J software and data were normalised to the loading control antibodies on each membrane. The fold change in phosphorylation compared to the 0 mM lactic acid vehicle controls was subsequently calculated.

### Western blotting

Cells were plated into six-well plates at a density of 5 × 10^5^ cells per well and incubated for 24 h, before media were replaced with DMEM (0 h) supplemented with glucose (5.6 mM), glutamine (2 mM), 10% FBS, lactic acid (0 or 10 mM) and uprosertib (10 μM) or vehicle (0.1% DMSO). After 1 h, media were aspirated from wells and cells were washed with 1x PBS. On ice, RIPA lysis buffer (Sigma-Aldrich, St. Louis, Missouri, US) containing 1x Halt Protease and Phosphatase Inhibitor Cocktail (Thermo Fisher Scientific, Waltham, Massachusetts, US) was added to wells, which were scraped to extract whole-cell protein lysates. Protein quantification was determined using the Pierce Bicinchoninic Acid Protein Assay Kit (Thermo Fisher Scientific, Waltham, Massachusetts, US) according to the manufacturer’s protocol. Cell lysates containing 15 μg of protein were loaded onto 4–20% Mini-PROTEAN® TGX^TM^ Precast Protein Gels (Bio-Rad, Hercules, California, US) and separated by sodium-dodecyl sulphate-polyacrylamide gel electrophoresis before being transferred to nitrocellulose membranes. Membranes were incubated using selected antibodies and chemiluminescent signals were enhanced using the Supersignal^TM^ West Pico plus chemiluminescent substrates (Thermo Scientific, Rockford, US) and detected on Amersham Hyperfilm^TM^ ECL films (GE Healthcare, Chicago, Illinois, US) using the Optimax X-ray film processor (Protec Healthcare, Oberstenfeld, Germany). Densitometry was performed using Image-J software. Data were normalised to the relevant total protein and subsequent fold change in phosphorylation compared to the 0 mM lactic acid vehicle control was calculated. The following primary antibodies were used: Pan Akt (C67E7, #4691), phosphorylated Akt Serine (S) 473 (D9E, #4060), Phosphorylated PRAS40 Threonine (T) 246 (#2640) and total PRAS40 (#2610) purchased from Cell Signalling Technologies (Danvers, Massachusetts, US). Anti-β-actin (#A5441) was purchased from Sigma-Aldrich (St. Louis, Missouri, US).

### ^1^H nuclear magnetic resonance (NMR) spectroscopy

Cells were plated into 12-well plates at a density of 1 × 10^5^ cells per well and incubated for 24 h, before media were replaced with DMEM (0 h) supplemented with glucose (5.6 mM), glutamine (2 mM), 10% FBS, lactic acid (0 or 10 mM) and uprosertib (10 μM) or vehicle (0.1% DMSO). After a further 24 h, media were collected from wells into microcentrifuge tubes and centrifuged at 150 *× g* for 5 mins. A volume of 550 μL of each media sample was transferred to a clean microcentrifuge tube. Subsequently, 50 μL of the internal calibration standard 4-4-dimethyl-4-silapentane-1-sulfonic acid in deuterium oxide (12 mM) was added before tubes were vortexed and centrifuged at 20,000*×g* for 1 min. Samples were transferred into 5 mm diameter NMR economy sample tubes (Wilmad-LabGlass, New Jersey, US).

High-resolution 1-dimensional ^1^H NMR spectroscopy was performed using the 14.1 T Bruker AVANCE 400 MHz spectrometer (Bruker BioSpin, Billerica, Massachusetts, US) at 298 K. NMR spectra were acquired using a conventional ZGPR solvent pre-saturation method with a single radiofrequency pulse, a recycle delay (d1) of 4 s, spectral width of 6402.049 Hz, 32 free induction decays and 64,000 data points. Data were automatically Fourier-transformed before being processed in MATLAB® software (Mathworks) using in-house scripts developed by J.T. Pearce, H.C. Keun, T.M.D. Ebbels and R. Cavill at Imperial College London (London, UK). Phase correction, baseline correction and normalisation to the internal standard reference peak was automatically done before spectral peaks were identified with reference to the Human Metabolome Database.

The rate of metabolite uptake and release was determined by calculating the difference in metabolite concentration (X) in spent medium compared to the initial medium. These values were subsequently normalised to the cell number obtained (area under the curve) using the Vi-Cell XR cell viability analyser, to give the rate in fmol/cell/hour. Negative values were converted to positive values and referred to as metabolite uptake.

$${\mathrm{Metabolite}}\,{\mathrm{uptake}}\,{\mathrm{and}}\,{\mathrm{release}} = \frac{{\left[ {\mathrm{X}} \right]{\mathrm{spent}} - \left[ {\mathrm{X}} \right]{\mathrm{initial}}}}{{{\mathrm{Area}}\,{\mathrm{under}}\,{\mathrm{the}}\,{\mathrm{curve}}}}$$

### Stable isotope labelling experiments by gas chromatography-mass spectrometry (GC-MS)

HCT116 and LS174T cells were seeded into six-well plates at a density of 6 × 10^5^ cells per well and incubated for 24 h. For glucose-labelling experiments media were supplemented with glutamine (2 mM), lactic acid (10 mM) and ^13^C_6_-glucose (5.6 mM, Sigma-Aldrich, Missouri, US). For lactic acid-labelling experiments media were supplemented with glutamine (2 mM), glucose (5.6 mM) and ^13^C_3_-lactic acid (10 mM, Sigma-Aldrich, Missouri, US). Media were also supplemented with uprosertib (10 μM) or vehicle (DMSO, 0.1%). Cells were treated for 4 h before intracellular metabolites were extracted and aqueous fractions were analysed using the Agilent 7890 GC system linked to an Agilent 5975 Mass Selective Detector using methods published previously.^26^ AMDIS software was used with reference to the NIST mass spectral library^27^ to identify metabolites. Peak integration was done using in-house developed GAVIN^28^ scripts for MATLAB® (MathWorks).

### Respiration measurements

To measure the oxygen consumption rate (OCR), LS174T cells were seeded at a density of 1 × 10^5^ cells per well into 96-well plates and incubated for 24 h. Cells were subsequently dosed in media (90 μL) supplemented with uprosertib (10 μM) and metformin (10 mM) for 2 h, before 10 μL of the MitoXpress Xtra Oxygen Consumption reagent (Agilent Technologies, Santa Clara, California, US) was added to each well according to the manufacturer’s instructions. Positive controls containing carbonyl cyanide-4 (trifluoromethoxy) phenylhydrazone (FCCP; 1.25 μM) were used. Wells were rapidly sealed with a layer of pre-warmed mineral oil and the plate was immediately read using the CLARIOstar plate reader at 37 °C (BMG Labtech, Ortenberg, Germany) in continuous cycles for 2 h. Fluorescence lifetime (μs) was calculated using the CLARIOstar MARS data analysis software (BMG Labtech, Ortenberg, Germany). OCR (μs, hour) was determined by calculating the maximum slope of the fluorescent lifetime.

### Statistical analysis

Statistical significance (*p < 0.05; **p < 0.01; ***p < 0.001 or ^#^*p* < 0.05; ^##^*p* < 0.01; ^*###*^*p* < 0.001) comparing groups with two independent variables was calculated using two-way ANOVA with Bonferroni’s multiple comparisons post hoc test. Statistical significance comparing three or more independent groups was calculated using one-way ANOVA with Bonferroni’s multiple comparisons post hoc test. Statistical significance (*p < 0.05; **p < 0.01; ***p < 0.001) comparing two independent groups was calculated using the Student’s *t* test. Calculations were performed and graphs were plotted using GraphPad Prism software version 8.10.

## Results

### Lactic acidosis induces resistance to uprosertib in colon cancer cell lines

**Fig. 1:**
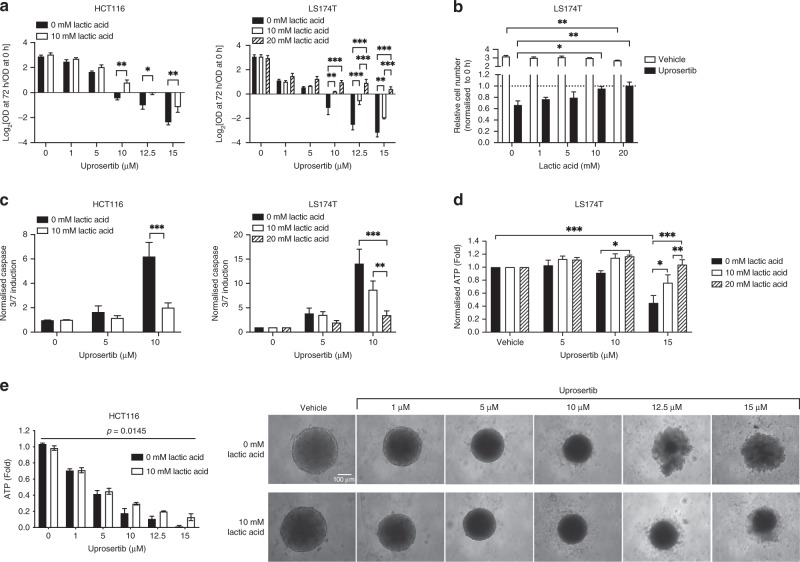
Lactic acid induces resistance to the pan-Akt inhibitor uprosertib in colon cancer cells. **a**, **b** Effects of uprosertib on survival in the presence or absence lactic acid. HCT116 and LS174T cell lines were treated for 72 h with uprosertib (1 μM to 15 μM) in the presence or absence of lactic acid (0–20 mM) and biomass was determined using SRB assays (**a**). LS174T cells were treated with uprosertib (10 μM) for 72 h before cells were counted (**b**). DMSO (0.1%) was used as a vehicle control. The results shown are normalised to the relative 0 h controls. **c** The effect of uprosertib on apoptosis in the presence or absence of lactic acid. Cells were treated for 24 h with uprosertib (5 or 10 μM) in the presence or absence of lactic acid (10 or 20 mM) and apoptosis was measured using a Caspase-Glo 3/7 assay (**c**). Results are shown as caspase 3/7 induction relative to cell biomass measured using SRB and the relevant vehicle controls. **d** The effect of uprosertib treatment (5, 10 and 15 μM) on ATP levels in the presence or absence of lactic acid in LS174T cells. Results are shown as ATP levels normalised to cell biomass measured using SRB and to the relevant vehicle controls. **e** Effect of uprosertib treatment and lactic acid on 3-D spheroids. HCT116 spheroids were dosed with uprosertib (1–15 μM) in the presence or absence of lactic acid (10 mM) for 72 h. Spheroid viability was quantified using a CellTitre-Glo 3-D assay and representative images of spheroids from one independent experiment are shown (**e**). The scale bar represents 100 μm. Quantified results are shown as fold change in ATP normalised to the relevant vehicle control. The dotted line at *y* = 1 in **b** indicates the initial 0 h cell number. The results shown are the mean ± SEM from three independent experiments (*n* = 3). *p < 0.05, **p < 0.01 and ***p < 0.001.

SRB cytotoxicity assays were used to determine the dose-response to uprosertib (1–15 μM) in the presence or absence of lactic acid (0, 10 or 20 mM) in HCT116 and LS174T cells after 72 h of treatment (Fig. 1a). Results were presented as Log_2_ of the OD at 72 h normalised to the 0-h OD to determine the cytotoxic or cytostatic effects of uprosertib treatment. Adding 20 mM of exogenous lactic acid reduced growth of HCT116 cells (Fig. S1), therefore this concentration was not used for further investigation of this line.

Uprosertib induced dose-dependent cytotoxicity in both cell lines in the absence of lactic acid. Lactic acid supplementation mediated a significant increase in the relative cell growth compared to conditions in the absence of lactic acid. Specifically, a cytostatic response to uprosertib in the presence of lactic acid was observed. Increasing the concentration of lactic acid increased the resistance to uprosertib, which was also demonstrated using cell counts (Fig. 1b).

To investigate further the role of lactic acid in protecting cells from the cytotoxic effects of uprosertib, apoptosis was measured using a Caspase-Glo 3/7 assay after 24 h of treatment (Fig. 1c). Results confirmed that uprosertib induced a dose-dependent increase in caspase-3/7 activation in HCT116 and LS174T cell lines, however, lactic acid mediated a significant reduction in caspase-3/7 activity. In LS174T cells, the higher the lactic acid concentration, the lower the caspase-3/7 activity; thus, lactic acid was associated with reduced apoptosis induction in cells treated with uprosertib. Uprosertib treatment in the presence of lactic acid was also associated with a significant increase in ATP levels (Fig. 1d). Overall, these data indicate that lactic acidosis induces resistance to uprosertib by promoting survival and a switch from a cytotoxic to a cytostatic response to treatment. Similar results were also observed in the presence of pyruvic acid (Fig. S2), which is another monocarboxylate that is transported via MCTs.^12,13^

Supplementing media with lactic acid was necessary for resistance to occur while supplementing with sodium lactate was insufficient (Fig. S3). Since lactic acid has a pKa of 3.86, it will dissociate into the lactate anion and H^+^ in media, lowering the pH (to 6.8 at 10 mM lactic acid, Fig. S4a). Lowering the extracellular pH to 6.5 alone using hydrochloric acid in the absence of lactate was not sufficient for protection against the cytotoxic effects of uprosertib (Fig. S4b).

### Lactic acidosis induces resistance to uprosertib in HCT116 3-D spheroids

It is well established that 3-D culture models better recapitulate the tumour microenvironment than monolayer culture by promoting the natural formation of pH, oxygen and nutrient gradients, for example ref. ^29^. We therefore used 3-D spheroids to investigate the response to uprosertib in the presence or absence of lactic acid (10 mM). Since LS174T cells did not readily form tight spheroids (data not shown), they were not investigated using this method.

A dose-dependent reduction in spheroid viability and size occurred upon treatment with uprosertib (Fig. 1e), however, spheroid viability was elevated in conditions with lactic acid supplementation compared to spheroids treated in the absence of lactic acid (*p* = 0.0145). Visualisation of spheroid morphology showed that spheroid integrity was better maintained at higher doses of uprosertib (12.5 and 15 μM) in the presence of lactic acid compared to spheroids treated in the absence of lactic acid. These data support the conclusion that lactic acidosis is associated with resistance and enhanced viability of colon cancer cells treated with uprosertib.

### Uprosertib treatment decreases downstream Akt signalling irrespective of the presence or absence of lactic acid

**Fig. 2:**
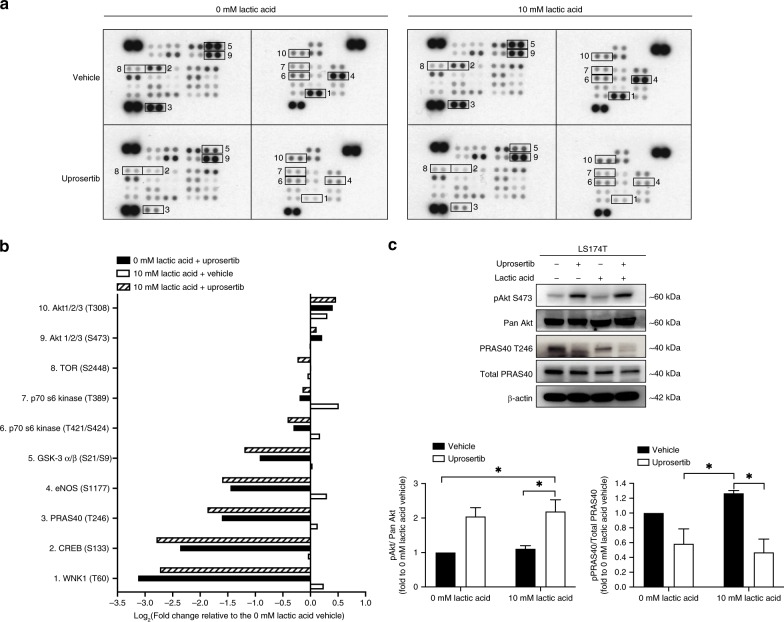
Uprosertib inhibits Akt signalling in the presence and absence of lactic acid. **a**–**c** Effect of uprosertib treatment in the presence or absence of lactic acid on Akt signalling. The proteome profiler human phosphokinase array kit was used to investigate the phosphorylation of downstream Akt signalling proteins in LS174T cells dosed with uprosertib (10 μM) for 1 h in the presence or absence of lactic acid (10 mM) (**a**). DMSO (0.1%) was used as a vehicle control. Densitometry using Image-J was done to quantify the expression of relevant phosphoproteins and results are shown as the Log_2_ of the fold change in phosphorylation compared to the 0 mM lactic acid vehicle controls (mean of technical duplicates) (**b**). Numbers on the array correspond to the numbered protein in the graph. Phosphorylation and total Akt and PRAS40 expression were examined using western blotting in LS174T cells after 1 h of uprosertib treatment (10 μM) in the presence or absence of lactic acid (10 mM) (**c**). β-actin was used as a loading control. Blots shown are from one representative experiment from a minimum of three independent replicates. Densitometry was performed to quantify expression of phospho-Akt and phospho-PRAS40. Data were normalised to the relevant total protein and graphs were plotted as fold change relative to the 0 mM lactic acid vehicle control. Data were presented as mean ± SEM from three independent replicates (*n* = 3). *p < 0.05.

Studies have demonstrated that uprosertib treatment leads to reduced phosphorylation of multiple proteins downstream of Akt, as well as an increase in feedback phosphorylation of Akt itself.^19,20^ Therefore, we investigated whether downstream inhibition of Akt signalling induced by uprosertib was altered in the presence of lactic acid. We used a human phosphokinase array to examine the expression of multiple phosphoproteins in LS174T cells after 1 h of uprosertib treatment (10 μM) in the presence or absence of lactic acid (10 mM) (Fig. 2a). Densitometry was performed and data were expressed as the mean fold change in phosphorylation relative to the 0 mM lactic acid vehicle control (0.1% DMSO) (Fig. 2b).

Uprosertib treatment in the absence of lactic acid caused a reduction in phosphorylation of several proteins downstream of Akt activation including GSK-3α/β (S21/S9), PRAS40 (T246), CREB (S133) and WNK1 (T60). Further, a moderate reduction in p70 s6 kinase phosphorylation (T421, S424 and T389), no change in TOR phosphorylation (S2446) and increased Akt phosphorylation at both T308 and S473 were observed, which corresponds with previous reports of the consequences of uprosertib treatment.^19^ Lactic acid supplementation was associated with a slight increase in phosphorylation of Akt (T308), as well as several other downstream phosphoproteins including p70 s6 kinase (T421, S424 and T389), eNOS (S1177), WNK1 (T60) and PRAS40 (T246). Despite this, uprosertib treatment produced the same alterations to protein phosphorylation regardless of exogenous lactic acid supplementation. Western blotting subsequently validated that uprosertib treatment increased phosphorylation of Akt (S473) and decreased PRAS40 (T246) phosphorylation in the presence and absence of lactic acid (Fig. 2c). LC-MS/MS analysis also confirmed that cellular uptake of uprosertib was the same in the presence and absence of exogenous lactic acid (Fig. S5). Overall, these data indicate that uprosertib-induced changes in Akt signalling are unaffected by the presence of lactic acid.

### Uprosertib inhibits glucose uptake and utilisation

**Fig. 3:**
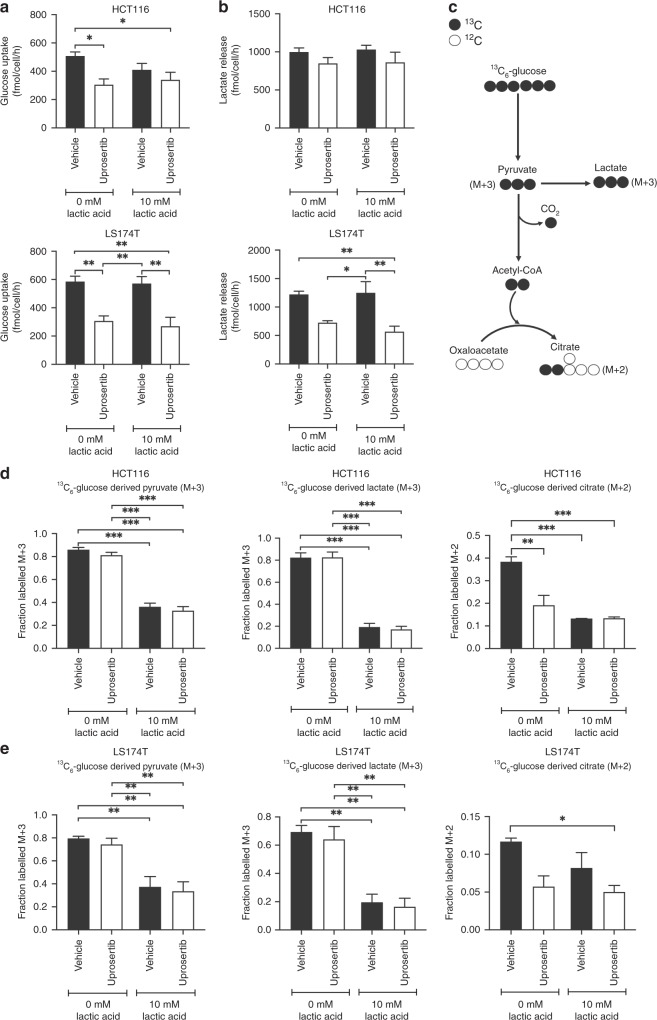
Uprosertib inhibits glucose utilisation in the presence and absence of lactic acid. **a**, **b** Effect of uprosertib and lactic acid on glucose uptake and lactate release rates. LS174T and HCT116 cells were treated with uprosertib (10 μM) in the presence or absence of lactic acid (10 mM) for 24 h before media were collected and cells counted. Extracellular glucose and lactate concentrations were determined using NMR spectroscopy and the rate of glucose uptake (**a**) or lactate release (**b**) in fmol/ cell/ hour was calculated. **c** Schematic representation of enrichment of ^13^C derived from ^13^C_6_-glucose into glycolytic and TCA cycle intermediates. **d**, **e** Effect of uprosertib treatment in the presence or absence of lactic acid on glucose utilisation in HCT116 (**d**) and LS174T (**e**) cells. Fraction labelled from ^13^C_6_-glucose into pyruvate (M + 3), lactate (M + 3) and citrate (M + 2) in the presence or absence of lactic acid (10 mM) after 4 h of treatment with uprosertib (10 μM). The results shown are the mean ± SEM from three independent experiments (*n* = 3). *p < 0.05, **p < 0.01 and ***p < 0.001.

Reduced glucose uptake is a pharmacodynamic marker of uprosertib treatment,^23^ therefore, we investigated how glucose uptake and utilisation was influenced by uprosertib (10 μM) in the presence and absence of lactic acid (10 mM) using ^1^H NMR spectroscopy (Fig. 3).

In the absence of lactic acid supplementation, uprosertib treatment caused a significant reduction in the rate of glucose uptake in both lines (40% and 48% reduction in HCT116 and LS174T cells respectively, Fig. 3a). Uprosertib treatment in the presence of lactic acid reduced the rate of glucose uptake in both lines to that observed with treatment in the absence of lactic acid. The reduction in glucose uptake in the presence of lactic acid and uprosertib was significant in LS174T cells. Uprosertib treatment in the presence of lactic acid also caused reductions in the rate of lactic acid release in LS174T cells (Fig. 3b). Lactic acid supplementation alone did not significantly alter the glucose uptake rate or lactate release rate in either line.

Since previous reports have indicated that lactate utilisation stimulates oxidative glutaminolysis,^30^ we also examined glutamine uptake in cells treated with uprosertib in the presence or absence of lactic acid (Fig. S6a, b). Uprosertib and lactic acid caused a modest reduction in the glutamine uptake rate in both lines (12–46% in HCT116 and 8–17% in LS174T cells), although these differences were not statistically significant.

To further investigate how glucose utilisation was influenced by uprosertib and exogenous lactic acid, stable isotope labelling using ^13^C_6_-glucose was used to trace the fate of glucose carbons after 4 h of treatment (Fig. 3c). Uprosertib treatment regardless of lactic acid did not change the ^13^C labelling pattern of pyruvate or lactate in both HCT116 (Fig. 3d) and LS174T (Fig. 3e) cells. However, labelling into citrate M + 2 was reduced significantly by uprosertib (50%) in the absence of lactic acid in both lines, consistent with reduced contribution of glucose carbon into the TCA cycle via pyruvate dehydrogenase (PDH) flux. In untreated HCT116 and LS174T cells, lactic acid supplementation decreased the relative abundance of M + 3 isotopologues of pyruvate and lactate and reduced the proportion of citrate M + 2, consistent with unlabelled lactate diluting out label contributions from ^13^C glucose to pyruvate. Under these conditions no subsequent effect of uprosertib on citrate M + 2 enrichment was observed in HCT116 cells and only a minor reduction in the LS174T cell line.

These data indicate that while uprosertib can inhibit glycolysis irrespective of lactic acid supplementation, in the absence of lactic acid uprosertib also limits glucose-derived pyruvate entry into the TCA cycle via PDH. By contrast, during lactic acidosis, the contribution of glucose to citrate production is lower and is not significantly reduced further by uprosertib compared to vehicle.

### The metabolic fate of exogenous lactic acid is unaffected by uprosertib treatment

**Fig. 4:**
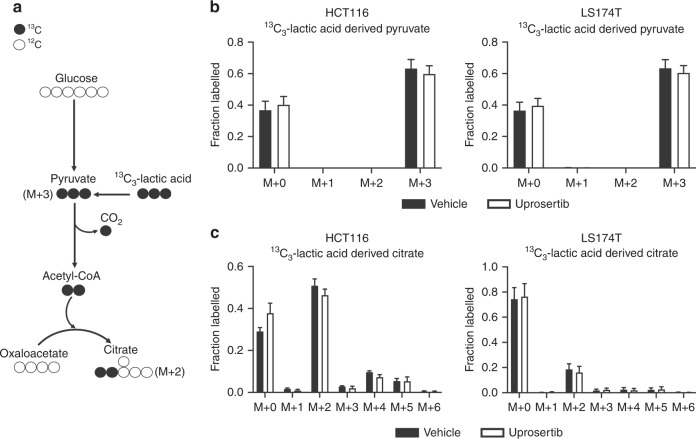
Enrichment of ^13^C_3_-lactic acid into cells is maintained in the presence of uprosertib. **a** Schematic representation of ^13^C_3_-lactic acid enrichment into pyruvate and TCA cycle intermediates. **b**, **c** Mass isotopologue distributions of ^13^C_3_-lactic acid enrichment into pyruvate (**b**) and citrate (**c**) after 4 h of incubation in the presence of uprosertib (10 μM) in LS174T and HCT116 cell lines. Cells were incubated in 10 mM of ^13^C_3_-lactic acid. DMSO (0.1%) was used for vehicle controls. The results shown are the mean ± SEM from three independent experiments (*n* = 3). *p < 0.05, **p < 0.01 and ***p < 0.001.

As lactic acid supplementation appeared to change glucose metabolism and the effect of uprosertib treatment, we tested whether exogenous lactate uptake and metabolism could be detected in these conditions. To investigate this, we used ^13^C_3_-lactic acid to trace the fate of lactate carbons in cells treated with uprosertib for 4 h before intracellular metabolites were extracted and analysed using GC-MS (Fig. 4a). Mass isotopologue distributions (MIDs) show that despite net production of lactate, ^13^C from exogenous lactic acid contributed significantly to the generation of pyruvate M + 3 and citrate M + 2 in both lines (Fig. 4b, c). Significantly higher enrichment of ^13^C_3_-lactic acid into pyruvate M + 3 and citrate M + 2 compared to ^13^C_3_-sodium lactate occurred in LS174T cells (Fig. S7) consistent with prior reports that lowering pH enhances ^13^C-lactate uptake and TCA cycle entry.^31^ This observation may also explain why sodium lactate in the absence of acidosis was not protective against uprosertib. Upon uprosertib treatment no significant alteration to the incorporation of ^13^C_3_-lactic acid carbon into pyruvate or citrate was observed. These data, together with our ^13^C glucose-labelling experiments, suggest that lactic acid supplementation decouples the TCA cycle from glycolysis in both cell lines tested and that the metabolism of exogenous lactate is unaffected by treatment with uprosertib.

Since the co-factor NAD^+^ is necessary for lactate dehydrogenase to catalyse the conversion of lactate to pyruvate, the NAD^+^/NADH ratio was determined. No significant changes in the NAD^+^/NADH ratio in the presence of lactic acid or uprosertib treatment were observed in HCT116 (Fig. S8a) or LS174T cells (Fig S8b), although a moderate reduction in HCT116 cells occurred.

### Inhibiting MCTs or oxidation is sufficient to rescue uprosertib-induced apoptosis in cancer cells exposed to lactic acidosis

**Fig. 5:**
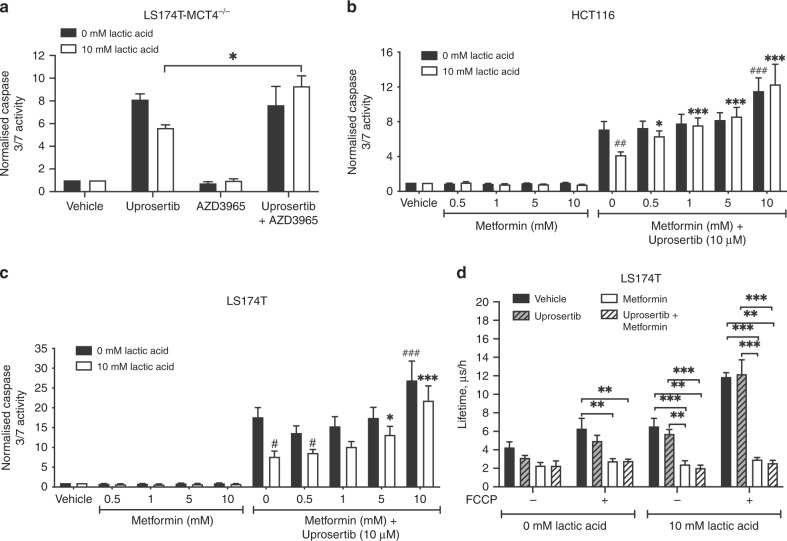
Targeting lactate transport and oxidative metabolism re-sensitises cells to uprosertib in the presence of lactic acid. **a** MCT1 inhibition using AZD3965 in combination with uprosertib in LS174T-MCT4^−/−^ cells. LS174T-MCT4^−/−^ cells were treated with uprosertib (10 μM) in combination with AZD3965 (1 μM) for 24 h (**a**). Apoptosis was measured using the Caspase-Glo 3/7 assay. Results were normalised to cell density measured using SRB assays and also to the vehicle controls. The results shown are the mean ± SEM from five independent experiments (*n* = 5). **b**, **c** The effect of combining metformin with uprosertib on apoptosis. HCT116 (**b**) and LS174T (**c**) cell lines were dosed with metformin (0.5 mM to 10 mM) alone or in combination with uprosertib (10 μM) for 24 h before apoptosis was measured using a Caspase-Glo 3/7 assay. Results are shown as caspase 3/7 induction relative to cell biomass measured using SRB and also to the relevant vehicle controls. **d** The effect of metformin and uprosertib treatment on the OCR in the presence and absence of lactic acid. LS174T cells were treated with metformin and uprosertib in the presence and absence of lactic acid for 2 h (**d**). FCCP was used as a positive control to measure the maximum respiratory capacity. The MitoXpress Xtra reagent was added to wells before fluorescence was measured over 2 h. The OCR was calculated from the slope of the fluorescent lifetime calculated using MARS analysis software. Graphs were plotted as OCR (lifetime, μs/h). The results shown are the mean ± SEM from four independent experiments (*n* = 4*)*. In **a**, **d** statistical significance is indicated by a line with *p < 0.05, **p < 0.01 or ***p < 0.001. In **b**, **c** statistical significance compared to the 0 mM lactic acid uprosertib only condition is denoted as ^#^*p* < 0.05, ^##^*p* < 0.01 or ^*###*^*p* < 0.001 and compared to the 10 mM lactic acid uprosertib only condition is denoted as *p < 0.05 or ***p < 0.001.

Since exogenous lactate contribution to the TCA cycle was maintained in cells treated with uprosertib, we hypothesised that inhibiting lactate uptake via MCTs or oxidation could re-sensitise cells to uprosertib in the presence of lactic acid. Thus cells were treated with uprosertib (10 μM) in combination with either the MCT1 inhibitor, AZD3965 (1 μM), or the mitochondrial complex I inhibitor metformin^32,33^ (0.5–10 mM) for 24 h, before assessing sensitivity using a Caspase-Glo 3/7 assay (Fig. 5). MCT1 and MCT4 are functionally redundant,^34^ therefore LS174T cells with MCT4^−/−^ status were used for experiments with AZD3965 treatment. MCT4 knockout was confirmed using western blotting (Fig. S9a).

Inhibition of lactate uptake using AZD3965 in LS174T-MCT4^−/−^ cells significantly rescued sensitivity to uprosertib in the presence of lactic acid (Fig. 5a). ^13^C_3_-lactic acid labelling confirmed AZD3965 treatment blocked lactate carbon entry into cells (Fig. S9b). In contrast, in LS174T-wild type cells, AZD3965 treatment did not block ^13^C_3_-lactic acid entry or rescue sensitivity to uprosertib in the presence of lactic acid (Fig. S9c, d).

Metformin as a monotherapy up to 10 mM did not induce apoptosis in either cell line (Fig. 5b, c). However, metformin treatment at the same doses in combination with uprosertib potentiated apoptosis in both lines irrespective of lactic acid supplementation. Notably in both lines, metformin treatment under lactic acidosis was sufficient to restore caspase-3/7 activity induced by uprosertib to levels observed in cells treated with uprosertib alone in the absence of lactic acid. The same rescue in sensitivity was observed using the Complex I and III inhibitors rotenone and antimycin A in both lines (Fig. S10), supporting the importance of OXPHOS in the protective effect of lactic acid.

To confirm that metformin was inhibiting respiration, the OCR was measured using the MitoXpress Xtra oxygen probe in LS174T cells (Fig. 5d). FCCP was used to measure the maximum oxygen consumption capacity. Results showed that metformin treatment reduced the OCR in all conditions tested, thus confirming that inhibiting oxidation is associated with enhanced sensitivity to uprosertib treatment. Furthermore, uprosertib treatment did not significantly alter the OCR, whereas lactic acid supplementation enhanced the basal OCR and significantly increased the maximum respiratory capacity of cells, as indicated in FCCP treated conditions. Overall, these data confirm that targeting mitochondrial oxidation using metformin, OXPHOS inhibitors or blocking lactate uptake can potentiate apoptosis and restore sensitivity to uprosertib treatment under lactic acidosis.

## Discussion

**Fig. 6:**
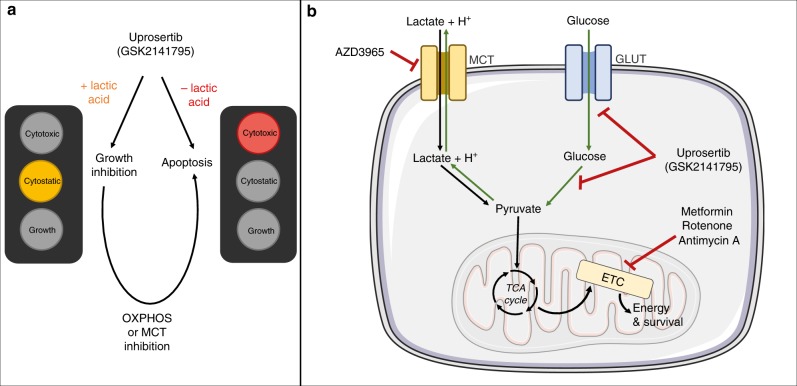
Schematic representation of the impact of uprosertib treatment combined with OXPHOS or MCT inhibition in cancer cells exposed to lactic acidosis. **a** Alteration of cellular fate in the presence or absence of lactic acid. **b** Schematic of the possible metabolic effects associated with altered response to treatment. Exogenous lactic acid decouples glycolysis from OXPHOS as lactate (black arrows) is preferentially utilised via the TCA cycle over glucose (green arrows). ETC electron transport chain. This figure was produced and adapted using Servier Medical Art licensed under the Creative Commons Attribution 3.0 Unported License (https://www.servier.com).

In the present study, we demonstrated that lactic acid supplementation is sufficient to induce resistance to the Akt inhibitor uprosertib in LS174T and HCT116 colon cancer cell lines and present evidence that metabolic factors may play a role (Fig. 6). Clinical reports indicate that uprosertib treatment as a monotherapy is primarily associated with tumour growth inhibition and stable disease at the recommended phase II dose (75 mg) in patients with solid tumours.^22^ Since lactate concentrations and acidity are often elevated in the tumour microenvironment, the cytostatic response to uprosertib in the presence of lactic acid in our experimental system potentially mimics the stable disease response that has been observed in patients.

Glycolysis inhibition occurred upon treatment with uprosertib in the presence and absence of lactic acid supplementation. Similarly, uprosertib treatment has been shown to cause dose-dependent hyperglycaemia and reduced glucose uptake in patients with gynaecological malignancies.^22,23^ Glucose carbon entry into the TCA cycle via PDH was also inhibited by uprosertib, consistent with previous work that suggests a direct interaction between PDH subunit B and AKT1 and that silencing of AKT1 could reduce PDH flux from ^13^C labelled glucose.^35^ By contrast exogenous lactate incorporation into citrate was maintained in cells treated with uprosertib. Furthermore, lactic acidosis stimulated an increase in respiration and respiratory capacity in our cell models. Thus, a decoupling of glycolysis from the TCA cycle and enhanced oxidative metabolism induced by lactic acid might be a mechanism of resistance to uprosertib.

The proportion of citrate M + 2 derived from ^13^C_3_-lactate was greater than that derived from ^13^C_6_-glucose, consistent with previous observations that isotope labelling from circulating lactate to the TCA cycle exceeds that from glucose in most tissues in vivo.^15,16^ Despite this, isotope exchange fluxes can lead to overestimations of the net contribution of lactate into the TCA cycle.^31^ Several models describing intracellular compartmentalisation of lactate/ pyruvate metabolism have been proposed, including the presence of intracellular mitochondrial shuttles for lactate oxidation;^36–38^ this may also explain our observations.

Several studies have previously described associations between altered tumour metabolism and treatment efficacy. Notably, oestrogen-related receptor alpha dependent lactate oxidation renders breast cancer cells resistant to phosphatidylinositol-3-kinase (PI3K) and mammalian target of rapamycin (mTOR) inhibitors cultured in the absence of glucose.^39^ Additionally, enhanced mitochondrial OXPHOS coupled with suppressed glycolysis is associated with therapeutic resistance to several other targeted therapies in cancer cells.^40–42^ Overall, these data combined with findings from the present report highlight the importance of metabolic flexibility in cancer cells in response to targeted therapies.

Clinical investigations combining uprosertib with the MEK inhibitor trametinib in several cancers types have been underway, however, similar studies in multiple myeloma, melanoma and cervical cancer have revealed no clinical benefits of this combination.^43–46^ Although uprosertib treatment as a monotherapy has been well tolerated,^22,23^ the adverse toxicity profile of phase II combination studies has been deemed unacceptable, thus, uprosertib is not currently under further development.^46^ Since we have demonstrated that enhanced OXPHOS in the presence of lactic acid is related to uprosertib resistance, and previous reports highlight that resistance to MEK combination therapies also corresponds with enhanced OXPHOS,^41^ this metabolic phenotype should be considered as a possible contributor to the lack of clinical efficacy observed during phase II trials with uprosertib. Thus, combining uprosertib with inhibitors of mitochondrial OXPHOS might present a valid therapeutic strategy to improve responses at tolerated doses. In support of this, metformin has been demonstrated to cause metabolic adaptations in human leukaemic cells that increases sensitivity to Akt inhibition.^47^

We observed that the higher the lactic acid concentration in media, the more resistant LS174T cells were to uprosertib and also that lactate addition or media acidification alone were insufficient for protection. MCTs are proton-dependent transporters,^48,49^ as a result, the direction of MCT function is dependent on the pH gradient across the membrane. Therefore, the requirement for exogenous lactic acid supplementation is consistent with enhanced lactate uptake being a necessary condition for uprosertib resistance. Such conditions have also been shown to promote reverse lactate dehydrogenase flux.^50^ Although we cannot rule out completely that intracellular acidosis contributes to the protective effect, combining uprosertib with the phase I clinical candidate MCT1 inhibitor AZD3965^51–53^ in MCT4 knockout cells, rescued sensitivity to uprosertib under lactic acidosis. This suggests that interference with lactate transport could also enhance tumour response to uprosertib in the clinic.

Uprosertib treatment induced apoptosis, which was significantly reduced in the presence of lactic acid. However, other PI3K/ Akt/ mTOR inhibitors tested did not stimulate apoptosis and no lactic acid-induced resistance was observed (Fig. S11, 12). Therefore, apoptosis and cytotoxicity appear to be necessary for lactic acid-induced resistance to occur. Alternative, high affinity targets of uprosertib,^20,21^ other than Akt, have been proposed, and it is possible that these interactions may drive the cytotoxic effect observed. However, a full investigation of off-target effects was beyond the scope of the current investigation.

Finally, although the present study has primarily considered lactate utilisation as a mechanism of resistance to uprosertib, lactic acidosis may also induce resistance through other mechanisms. For instance, lactate has been shown to act as an agonist for the G-protein coupled receptor 81, which upregulates expression of genes associated with lactate metabolism and survival.^54–56^ Lactate has also been demonstrated to increase expression of the anti-apoptotic protein Bcl-2 via the PI3K/ Akt/ mTOR signalling pathway, promoting survival under glucose deprivation.^57^ Additionally, the conversion of lactate to pyruvate by lactate dehydrogenase B has been shown to promote lysosomal acidification and autophagy, which is sufficient for survival under conditions of nutrient stress, such as glucose deprivation.^58^

In conclusion, we have demonstrated that lactic acidosis protects colon cancer cells from the cytotoxic effects of uprosertib treatment. Lactate uptake and enhanced oxidation in the presence of uprosertib was associated with resistance and inhibiting either re-sensitised cells to treatment. The results presented here highlight the potential influence of metabolic plasticity to mediate response to targeted therapy against oncogenic pathways that also regulate glucose metabolism. Further, our findings provide new insight into a potential mechanism of resistance to uprosertib, which may in part explain the limited efficacy that has been observed in clinical trials.

## Supplementary information

Supplementary material

## Data Availability

Data generated for the current study are available from the corresponding author on reasonable request.
