# Supplementary material for: Lactic acidosis induces resistance to the pan-Akt inhibitor uprosertib in colon cancer cells
Source: Br J Cancer. 2020 Mar 10;122(9):1298–308. doi: 10.1038/s41416-020-0777-y (PMC7188671; doi:10.1038/s41416-020-0777-y)
Supplement: Supplementary file 1 — Supplementary material [file 41416_2020_777_MOESM1_ESM.docx]

**­SUPPLEMENTARY METHODS**

**NAD/NADH-Glo assay**

To measure the levels of NAD and NADH in cells LS174T and HCT116 cell lines, the NAD/NADH-Glo^TM^ (Promega, Southampton, UK) bioluminescent assay was used according to the manufacturer’s protocol. Cells were plated at a density of 4 x 10^4^ and incubated for 24 hours before media were changed to test media containing glucose (5.6 mM), glutamine (2 mM), 10% FBS and lactic acid (0 mM or 10 mM). Cells were also dosed with uprosertib (10 μM) or DMSO (0.1%) before plates were analysed after 24 hours. The NAD^+^ signal was normalised to the NADH signal so graphs were plotted as the ratio of NAD^+^/ NADH.

**pH measurements**

The pH of DMEM was measured immediately after glucose (5.6 mM), glutamine (2 mM), 10% FBS or lactic acid (0, 10 or 20 mM) was added to media. To adjust media to pH 6.5, hydrochloric acid was added dropwise to DMEM containing glucose (5.6 mM), glutamine (2 mM) and 10% FBS until the pH measured 6.5. To measure pH, a Mettler Toledo InLab Routine Pro electrode was used.

**Intracellular uprosertib measurements using liquid-chromatography mass-spectrometry (LC-MS).**

A density of 8 x10^5^ cells per well were plated into 6-well plates and incubated for 24 hours before media were changed to DMEM (0 hours) supplemented with glucose (5.6 mM), glutamine (2 mM), 10% FBS and lactic acid (0 or 10 mM) with or without uprosertib (0 or 10 μM). Untreated and vehicle (0.1% DMSO) controls were also used. After 24 hours of treatment, media were aspirated, wells were washed twice with Ringer’s buffer and cells were quenched using ice cold methanol. Samples were subsequently dried under nitrogen gas, before the samples were deproteinised using 800 µL MeOH/H_2_O 3/1 v/v (LC-MS grade), incubated in -20 °C for 30 min and then centrifuged (8500 rcf for 10 min, 8°C). A volume of 700 µL of the supernatant was transferred into high recovery LC-MS vials and evaporated under nitrogen flow, before being reconstituted in 100 µL of AcCn/H_2_O 1/9 v/v (LC-MS grade).

LC-MS analysis was performed by an ultra-performance liquid chromatography system (1290 Agilent) hyphenated to a 4000 QTrap mass analyser (AB Sciex) equipped with a Turbo V electrospray source in the positive ionisation mode. Direct infusion of a 1 µg/mL uprosertib solution in AcCN/H_2_O 1/1 v/v with 0.1 % formic acid was performed to define the MRM transitions specific for uprosertib analysis and also to define the optimum DP and CE parameters for each MRM transition.

Reversed phase (RP) chromatographic separation was performed for the quantification of uprosertib. An Acquity HSS T3 C18 column (2.1 mm x 100 mm, 1.8 μm) at a flow rate of 0.6 mL/ min maintaining the temperature at 40°C and using 5 µL of injection volume. The mobile phase consisted of A (LC-MS grade H_2_O with 0.2% formic acid) and B (LC-MS grade ACN with 0.2% formic acid). The elution gradient profile was as follows (minute / % of B): 0/0.5, 2/0.5, 5/15, 10/99.5, 13/99.5, 13.1/0.5, 15/0.5). The following five MRM transition were recorded: (Q1/Q3 Masses *m/z*): 429.40/412.40 Da, 429.40/243.00 Da, 429.40/153.20 Da, 429.40/133.30 Da and 429.40/127.30 Da. The *m/z* 429.40/412.40 Da was used for quantification and the other four for confirmation. Each cell sample was run in duplicate and standards of uprosertib were run for quantification, identification and confirmation of retention time.

**Analysis of MCT4 protein expression using western blotting**

Protein extraction was performed as described in the main article. Cell lysates containing 40 μg of protein were loaded onto 10% Mini-PROTEAN^®^ TGX^TM^ Precast Protein Gels (Bio-Rad, Hercules, California, US) before being separated by gel electrophoresis and transferred onto nitrocellulose membranes. Membranes were incubated in anti-MCT4 ((H-90) #50329) or α-tubulin ((H-235) #9104) purchased from Santa Cruz Biotechnology (Dallas, Texas, US). Chemiluminescent signals were enhanced using West Pico plus chemiluminescent substrates (Thermo Scientific, Rockford, US) and detected using the ImageQuant LAS 4000 imager (GE Healthcare Life Sciences). Blots were analysed using Image Studio Lite (LI-COR software).

**SUPPLEMENTARY FIGURES**

**
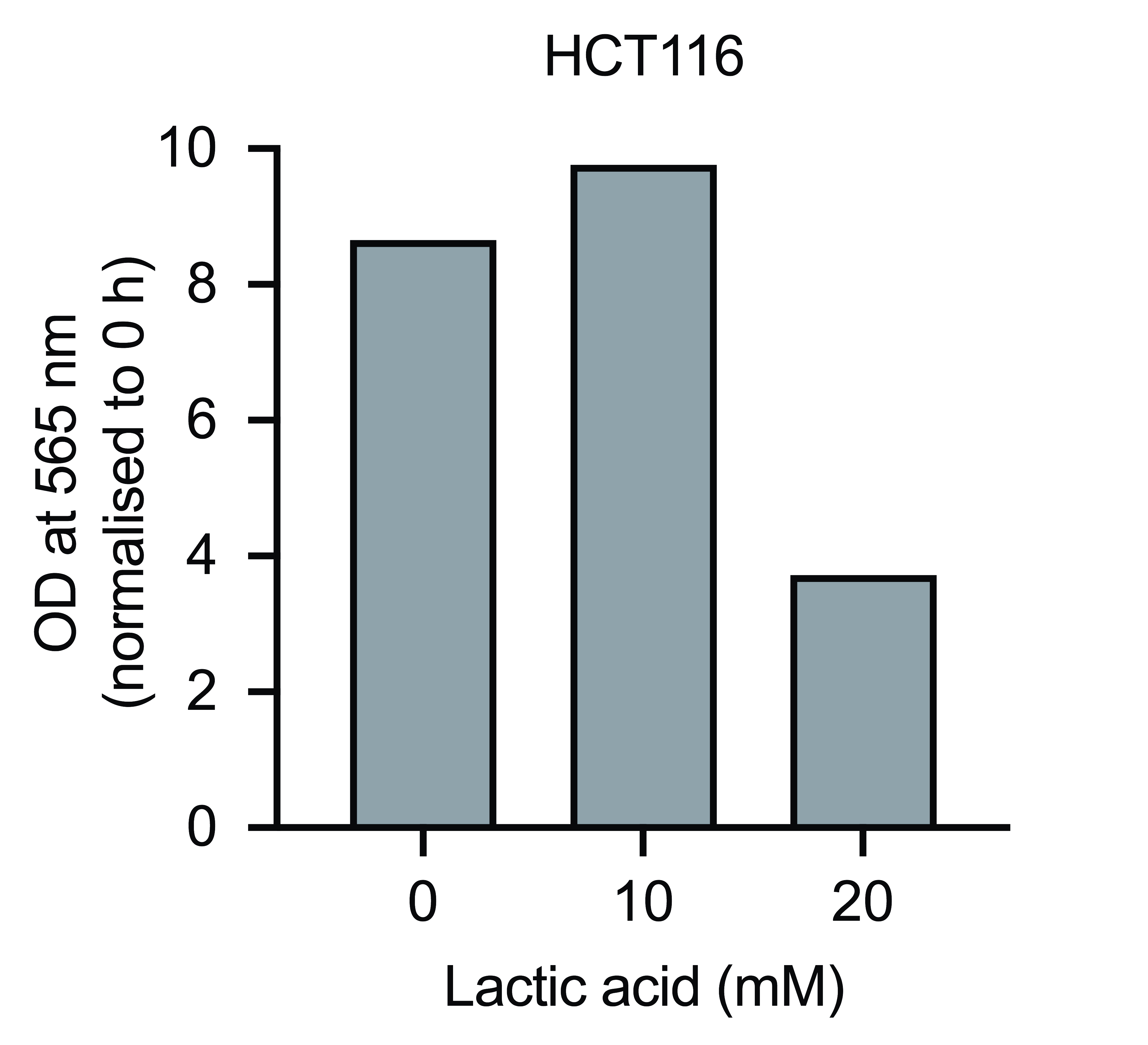
**

Fig. S1 The effect of lactic acid on growth of HCT116 cells

HCT116 cells were cultured for three days in DMEM supplemented with 0, 10 or 20 mM lactic acid for 72 hours. Cell density was determined using SRB assays and graphs were plotted as the OD at 72 hours normalised to the OD at 0 hours. The results shown are the mean of three technical replicates from one independent experiment (*n*=1).

Fig. S2 Uprosertib treatment in the presence of lactic acid or pyruvic acid

**a** The effect of lactic acid or pyruvic acid on cytotoxicity measured using SRB assays after 72 hours of uprosertib (10 μM) treatment in LS174T cells (**a**). **b, c** The effect of lactic acid or pyruvic acid on caspase 3/7 activity after 24 hours of uprosertib (10 μM) treatment in LS174T (**b**) and HCT116 (**c**) cells. **d, e** The effect of lactic acid or pyruvic acid on ATP levels in LS174T (**d**) and HCT116 (**e**) cells after 24 hours of uprosertib treatment (5 μM, 10 μM or 15 μM). Controls without lactic acid or pyruvic acid supplementation and vehicle controls containing DMSO (0.1%) were used for all experiments. Data were presented as mean ± SEM from three independent experiments in a, c, d and e (*n*=3), and from four independent experiments in b (*n*=4). **p*<0.05, ***p*<0.01 and ****p*<0.001. In b, statistical significance compared to the control in the same treatment condition is shown as ^###^*p*<0.001 above bars.

Fig. S3 Uprosertib treatment in the presence and absence of sodium lactate

**a**, **b** The effect of uprosertib (0.1 μM to 15 μM) treatment on HCT116 (**a**) and LS174T (**b**) cells in the presence or absence of sodium lactate (10 mM) after 72 hours was measured using SRB assays. Media were replenished every 24 hours to control for changes in the extracellular metabolite concentrations. DMSO (0.1%) was used for vehicle controls. The results shown are the mean ± SEM from three independent experiments (*n*=3).

Fig. S4 The effect of low pH on sensitivity to uprosertib.

**a** The pH of DMEM supplemented with glucose (5.6 mM), glutamine (2 mM), 10% FBS and lactic acid (0, 10 or 20 mM) was measured using a Mettler Toledo InLab Routine Pro electrode. **b** The effect of low pH induced by adding hydrochloric acid to DMEM supplemented with glucose (5.6 mM), glutamine (2 mM) and 10% FBS in LS174T cells after 72 hours of treatment with uprosertib (10 μM). Media were changed every 24 hours to control for changes in extracellular metabolite concentrations. SRB cytotoxicity assays were performed at 72 hours and the optical density was normalised to the optical density at 0 hours. DMSO (0.1%) was used for vehicle controls. The results shown are the mean ± SEM from three independent experiments (*n*=3). **p*<0.05 or ****p*<0.001.

Fig. S5 Intracellular uprosertib concentration in the presence and absence of lactic acid in HCT116 cells.

HCT116 cells were treated with uprosertib (10 μM) in the presence or absence of lactic acid (10 mM). Untreated and vehicle (0.1% DMSO) controls were also used. Intracellular material was extracted after 24 hours of treatment using methanol quenching and intracellular uprosertib levels were measured using LC-MS. The concentration of uprosertib was subsequently calculated and normalised to the cell number, so graphs were plotted as the intracellular uprosertib concentration per cell (μg/ cell). Data were presented as mean ± SEM from two biological replicates (*n*=2) from one independent experiment, except the vehicle controls which were presented as the mean from one biological replicate (*n*=1).

Fig. S6 The rate of glutamine uptake in the cells treated with uprosertib in the presence and absence of lactic acid.

**a**, , **b** HCT116 (**a**) and LS174T (**b**) cells were dosed with uprosertib (10 μM) for 24 hours in the presence or absence of lactic acid (10 mM). Media were collected and analysed using NMR spectroscopy. Cell counts at 0 hours and 24 hours were also obtained. The changes in the glutamine concentration were calculated and normalised to the cell number so graphs were plotted as the rate of glutamine uptake in fmol/ cell/ hour. The results shown are the mean ± SEM from three independent experiments (*n*=3).

Fig. S7 MIDs of pyruvate and citrate derived from ^13^C_3_-lactic acid and ^13^C_3_-sodium lactate

**a, b** MIDs of pyruvate and citrate derived from ^13^C_3_-lactic acid and ^13^C_3_-sodium lactate in LS174T cells. Cells were incubated in DMEM supplemented with glucose (5.6 mM) and glutamine (2 mM) with either ^13^C_3_-lactic acid (10 mM) or ^13^C_3_-sodium lactate (10 mM) for 1 hour. Intracellular metabolites were subsequently extracted and analysed using GC-MS. MIDs of pyruvate (**a**) and citrate (**b**) were plotted. Data were presented as mean ± SEM from two independent experiments (*n*=2). **p*<0.05 or ****p*<0.001.

Fig. S8 Effect of uprosertib on NAD^+^/NADH in the presence and absence of lactic acid.

**a**, **b** The effect of uprosertib (10 μM) in the presence or absence of lactic acid (10 mM) after 24 hours of treatment on NAD^+^/NADH. Levels of NAD^+^ and NADH were measured using the NAD/NADH-Glo^TM^ assay. The NAD^+^ signal was normalised to the corresponding NADH signal, so graphs were plotted as the ratio of NAD^+^/NADH. The results shown are the mean ± SEM from three independent experiments (*n*=3).

Fig. S9 Effect of AZD3965 treatment in LS174T-MCT4^-/-^ and wild-type LS174T cells.

**a** MCT4 protein expression in wild-type (-WT) and LS174T-MCT4^-/-^ cells. Alpha-tubulin was used for loading controls. Blots shown are from one experiment representative of three independent replicates. **b, c** Effect of MCT1 inhibition using AZD3965 (1 μM) on ^13^C_3_-lactic acid incorporation into LS174T-MCT4^-/-^ (**b**) and the LS174T-WT cells (**c**) after 2 hrs of incubation. Pyruvate M+3 and citrate M+2 were plotted. DMSO (0.1%) was used for vehicle controls. Data were presented as mean of two technical repeats from one independent experiment (*n*=1). **d** Effect of uprosertib (10 μM) treatment on caspase 3/7 activity in combination with AZD3965 (1 μM) in the wild-type parental LS174T cell line after 24 hours of treatment. Data were presented as mean ± SEM from three independent experiments (*n*=3). All conditions with uprosertib were significantly increased (p<0.001) compared to vehicle or AZD3965 only treated cells (not indicated on graph). All other significant differences were indicated by a line, ***p*<0.01 or ****p*<0.001.

Fig. S10 The OXPHOS inhibitors rotenone and antimycin A rescue sensitivity to uprosertib treated cells exposed to lactic acidosis.

**a-d** Rotenone and antimycin A treatment re-sensitise LS174T and HCT116 cells to uprosertib under lactic acidosis. LS174T (**a, b**) and HCT116 (**c, d**) cells were treated with rotenone (1 and 5 μM) or antimycin A (1 and 5 μM) alone or in combination with uprosertib (10 μM) in the presence or absence of lactic acid (10 mM). Caspase-Glo 3/7 assays were performed after 24 hours, before results were normalised to cell density measured using SRB assays and subsequently to the vehicle controls. Data were presented as mean ± SEM from three independent experiments (*n*=3). **p*<0.05 or ***p*<0.01.


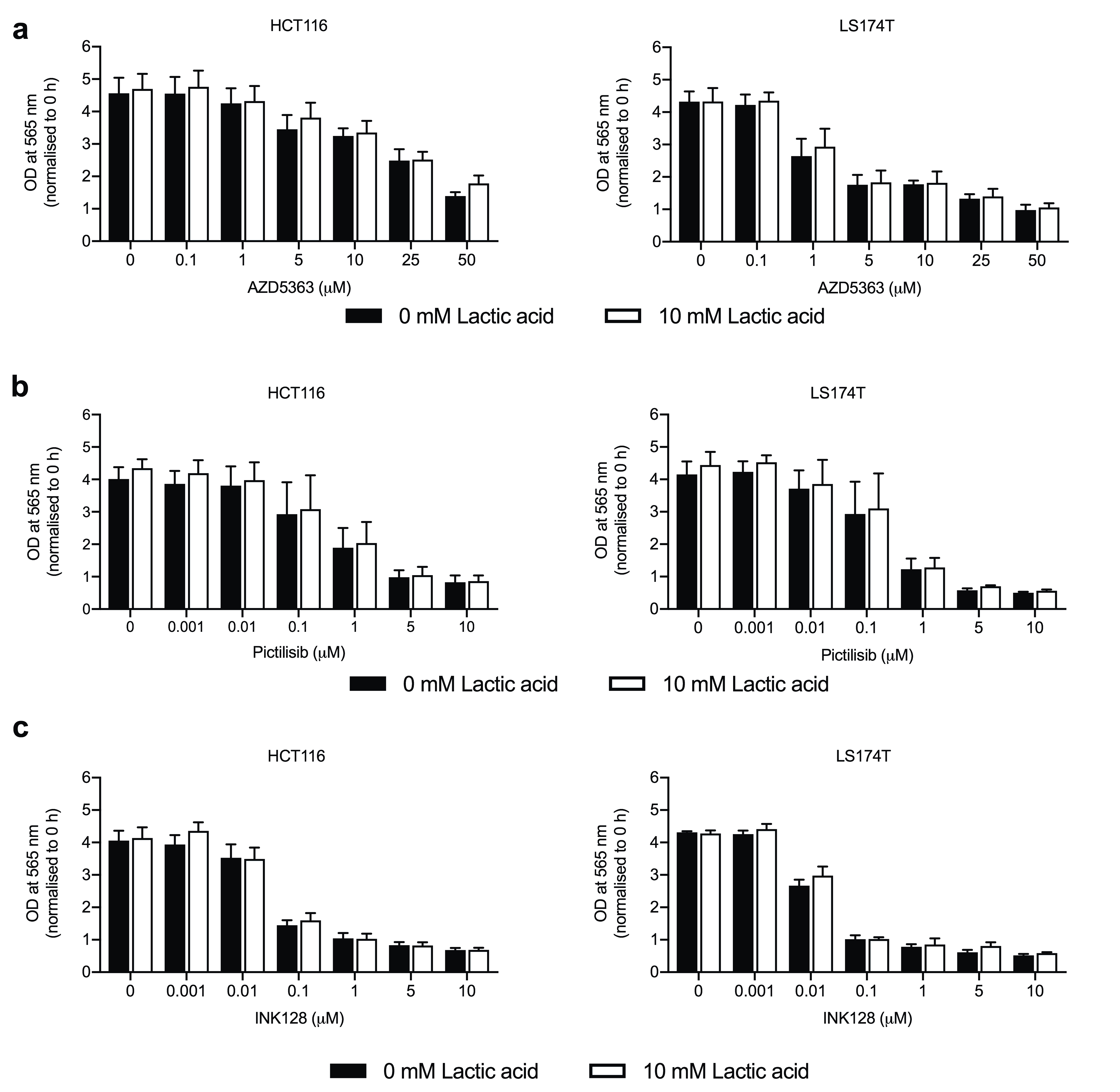


Fig. S11 Effect of lactic acid on response to PI3K/ Akt/ mTOR pathway inhibitors.

**a**-**c** LS174T and HCT116 cells were treated with AZD5363 (0.1 μM to 50 μM) (**a**), pictilisib (0.001 μM to 10 μM) (**b**) or INK128 (0.001 μM to 10 μM) (**c**) for 72 hours in the presence or absence of lactic acid (10 mM). SRB cytotoxicity assays were performed and results were normalised to the optical density at 0 hours. DMSO (0.1%) was used for vehicle controls. The results shown are the mean ± SEM from three independent experiments (*n*=3).

Fig. S12 The influence of PI3K/ Akt/ mTOR pathway inhibitors on apoptosis in the presence and absence of lactic acid.

**a**, **b** HCT116 (**a**) and LS174T (**b**) cells were treated with AZD3563 (1 μM and 5 μM), pictilisib (1 μM and 10 μM) or INK128 (1 μM and 10 μM) for 24 and 48 hours in the presence or absence of lactic acid (10 mM). Media were replenished every 24 hours to control for changes in the extracellular metabolite concentrations. Uprosertib (10 μM) was used as a positive control. DMSO (0.1%) was used for vehicle controls. The results shown are the mean ± SEM from three independent experiments (*n*=3). ***p*<0.01 or ****p*<0.001.
